# Elevated Incidence of Dental Caries in a Mouse Model of Cystic Fibrosis

**DOI:** 10.1371/journal.pone.0016549

**Published:** 2011-01-31

**Authors:** Marcelo A. Catalán, Kathleen Scott-Anne, Marlise I. Klein, Hyun Koo, William H. Bowen, James E. Melvin

**Affiliations:** 1 Department of Pharmacology and Physiology, University of Rochester Medical Center, Rochester, New York, United States of America; 2 Center for Oral Biology, University of Rochester Medical Center, Rochester, New York, United States of America; University of Liverpool, United Kingdom

## Abstract

**Background:**

Dental caries is the single most prevalent and costly infectious disease worldwide, affecting more than 90% of the population in the U.S. The development of dental cavities requires the colonization of the tooth surface by acid-producing bacteria, such as *Streptococcus mutans.* Saliva bicarbonate constitutes the main buffering system which neutralizes the pH fall generated by the plaque bacteria during sugar metabolism. We found that the saliva pH is severely decreased in a mouse model of cystic fibrosis disease (CF). Given the close relationship between pH and caries development, we hypothesized that caries incidence might be elevated in the mouse CF model.

**Methodology/Principal Findings:**

We induced carious lesions in CF and wildtype mice by infecting their oral cavity with *S. mutans*, a well-studied cariogenic bacterium. After infection, the mice were fed a high-sucrose diet for 5 weeks (diet 2000). The mice were then euthanized and their jaws removed for caries scoring and bacterial counting. A dramatic increase in caries and severity of lesions scores were apparent in CF mice compared to their wildtype littermates. The elevated incidence of carious lesions correlated with a striking increase in the *S. mutans* viable population in dental plaque (20-fold increase in CF vs. wildtype mice; *p* value<0.003; *t test*). We also found that the pilocarpine-stimulated saliva bicarbonate concentration was significantly reduced in CF mice (16±2 mM vs. 31±2 mM, CF and wildtype mice, respectively; *p* value<0.01; *t test*).

**Conclusions/Significance:**

Considering that bicarbonate is the most important pH buffering system in saliva, and the adherence and survival of aciduric bacteria such as *S. mutans* are enhanced at low pH values, we speculate that the decrease in the bicarbonate content and pH buffering of the saliva is at least partially responsible for the increased severity of lesions observed in the CF mouse.

## Introduction

Dental caries is the single most prevalent and costly infectious disease worldwide, affecting more than 90% of the population in the U.S. [1]. The development of dental cavities requires the colonization of the tooth surface by acid-producing bacteria, such as *Streptococcus mutans*, in conjunction with the frequent ingestion of a cariogenic high-sucrose diet, the substrate for acid and glucan production by organisms. The elevated amounts of acid and glucans modulate the establishment of cariogenic organisms within tightly adherent biofilms known as dental plaque. Numerous host-derived and dietary factors in saliva also affect the pathogenesis of this multifactorial disease [2]. A direct association between incidence of carious lesions and decreased saliva production is well documented [3], [4], [5], but the role of specific salivary constituents in the pathogenesis of dental caries is not well-understood, particularly in diseases such as cystic fibrosis (CF).

Cystic fibrosis is the most common genetic disease in Caucasians, occurring in approximately one out of 3,200 live births [6], and is generally associated with alterations in saliva composition [7], [8], [9], [10]. CF is caused by mutations of the *CFTR* (cystic fibrosis transmembrane conductance regulator) gene. CFTR is highly expressed in salivary glands [11], [12], [13], [14], [15], but the reported effects of CF on salivary gland function [6], [7], [8], [16], [17] and the incidence of dental cavities are inconsistent [16], [17], [18], [19], [20], [21]. The basis for these discrepancies is unknown, but many of these studies were performed before it was routine to determine the nature of the CF mutation, which relates to the severity of disease, and when patients rarely survived to adulthood because the treatment of CF was largely ineffective. Moreover, CF patients consume potentially anti-cariogenic foods such casein-containing dairy products [22], [23], [24] and are typically treated with wide-spectrum antibiotics, which alter the oral flora and likely mask the relationship of CF and caries production [16], [17].

To gain insight into the relationship between cystic fibrosis and incidence of dental caries we induced carious lesions in CF mice and their wildtype littermates. A dramatic increase in cavity formation and severity of lesions was apparent on both smooth and sulcal tooth surfaces in CF mice. The elevated incidence of carious lesions correlated with a dramatic increase in the *S. mutans* viable population in the plaque of CF mice, and a decrease in saliva pH and HCO_3_^-^ levels (associated with buffering capacity).

## Results

### Elevated incidence and severity of carious lesions in the ΔF508 mouse CF model

**Figure 1:**
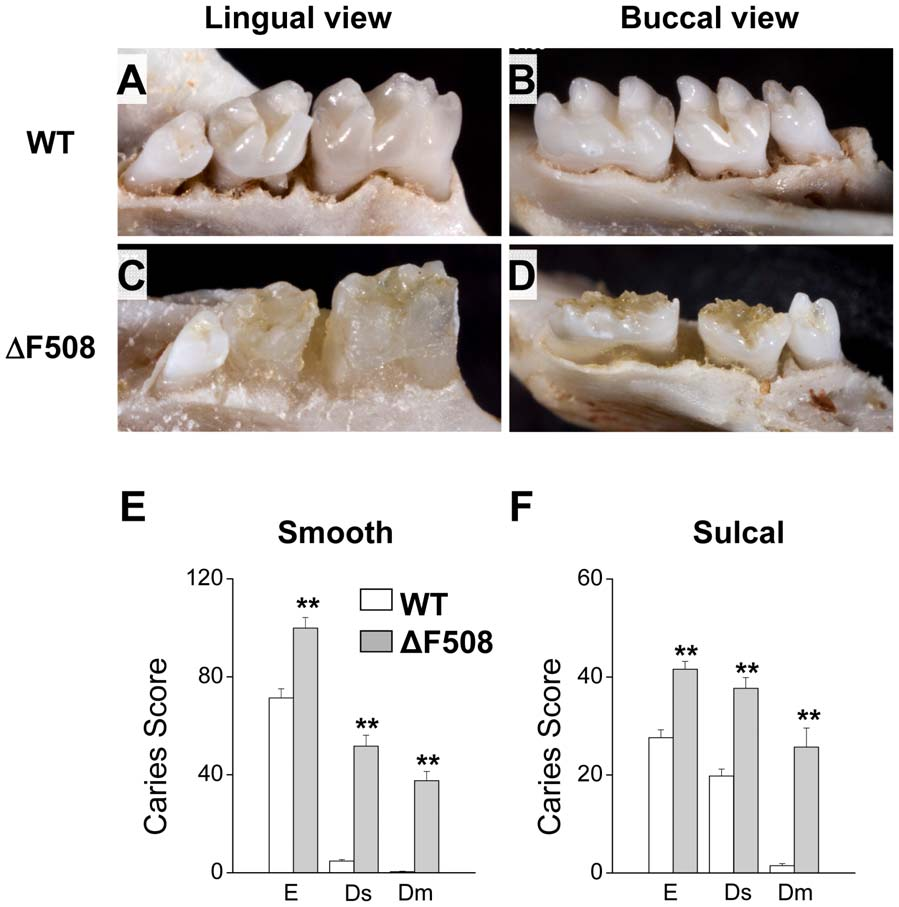
Elevated incidence and severity of carious lesions in ΔF508 mutant mice. **Panels A, B, C and D.** Lingual (panels A and C) and buccal (panels B and D) views of representative mandibular jaws from wildtype (WT, panels A and B) and mutant mice (ΔF508, panels C and D). **Panels E and F.** Smooth and sulcal caries and severity of lesions. Evaluations of carious enamel involvement in submandibular and maxillary first and second molars are expressed as E, while severities of carious lesions, based on degree of dentin involvement, are expressed as Ds and Dm (slight and moderate dentin involvement, respectively) [28]. Open and filled bars correspond to WT (n = 13) and ΔF508 (n = 7) mice, respectively. Values are given as the mean ± S.E.M. ** p<0.01, *t* test.

Of the more than 1,500 known disease-causing *CFTR* mutations, the most common mutation (∼90% of mutations) is a deletion of phenylalanine 508 (the ΔF508 mutation) [6]. To directly test the association between CF and dental caries we used the ΔF508 mouse CF model, which reproduces many of the defects observed in human disease [25]. A dramatic increase in number and severity of carious lesions was clearly evident after only five weeks exposure to a cariogenic diet (Figure 1). This was most apparent on the lingual and occlusal smooth surfaces where the enamel layer of the first and second mandibular and maxillary molars was nearly completely destroyed (compare mandibular molars of wildtype to ΔF508 mice, Figures 1A & 1B to 1C & 1D, respectively). Note that the relatively small third molars were essentially free of carious lesions. Mouse third molars typically erupt into the oral cavity 24 to 36 days postnatal [26], [27], after the cariogenic diet was introduced. The incidence and severity scores of the carious lesions for the first and second mandibular and maxillary molars of animals like those shown in Figures 1A-D are summarized in Figures 1E & 1F. The incidence of smooth surface and sulcal caries were elevated in mutant ΔF508 mice, while the increase in the severity of smooth surface and sulcal caries was especially dramatic (Figures 1E & 1F, respectively). Scores for carious enamel involvement are expressed as E, while severities of carious lesions, based on degree of dentin involvement, are expressed as Ds and Dm (slight and moderate dentin involvement, respectively) [28].

### Number of viable *S. mutans* are dramatically increased in the ΔF508 mouse CF model

**Figure 2:**
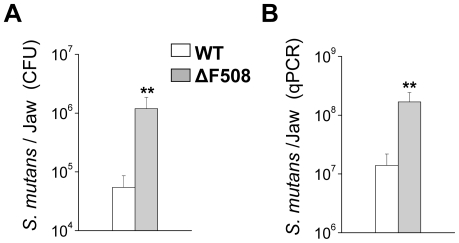
Number of viable *S. mutans* cells were dramatically increased in ΔF508 mutant mice. Bacterial counts in the lower jaws were evaluated by two independent techniques. **Panel A.** Numbers of colony forming units (CFU) were calculated by plating bacterial suspensions in MSB plates and counting using a grid plate system. **Panel B.** *S. mutans* in the mandibular jaws were also estimated using quantitative real-time PCR. Open and filled bars correspond to wildtype (WT, n = 9) and mutant (ΔF508, n = 5) mice, respectively. Values are given as the mean ± S.E.M. ** p<0.02, *t* test.

The higher number and severity of carious lesions observed in the ΔF508 mice suggest that the colonization of the tooth surface by acid-producing bacteria may have been enhanced in the CF mouse model. *S. mutans* is a critical microorganism associated with the pathogenesis of dental caries [2]. Figure 2A shows that the number of *S. mutans* colony forming units was ∼20 fold higher in the ΔF508 mice, correlating with the enhanced frequency and severity of caries lesions in these animals. Quantitative real-time PCR confirmed that the increase in the number of the caries-causing *S. mutans* population was more than an order of magnitude greater in the oral cavity of ΔF508 mice (Figure 2B).

### Saliva pH and bicarbonate concentration are dramatically reduced in the ΔF508 mouse CF model

**Figure 3:**
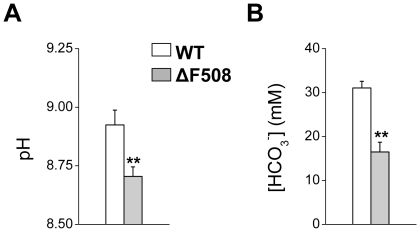
Saliva pH and bicarbonate concentration were dramatically reduced in ΔF508 mutant mice. Secretion was stimulated in non-infected mice by intraperitoneal injection of pilocarpine HCl (10 mg/kg). **Panel A.** pH value was measured immediately after saliva collection using a pH-sensitive electrode. **Panel B.** Bicarbonate was measured using an enzymatic-based colorimetric kit. Open and filled bars correspond to wildtype (WT, n = 7) and mutant (ΔF508, n = 6) mice, respectively. Values are given as the mean ± S.E.M. ** p<0.01, *t* test.

*S. mutans* is unusual in that its tight glucan-dependent adherence, growth and survival on tooth surfaces prefer a more acidic pH environment than most other oral microorganisms, consistent with its role in cavity formation [2]. Using the ΔF508 mouse cystic fibrosis model, we previously found that the CFTR channel mediates Cl^−^ reabsorption by salivary gland ducts [11]. We also noted that the pH of submandibular saliva was decreased in the ΔF508 mouse, suggesting that CFTR mutations may compromise the HCO_3_^−^ secretion mechanism. HCO_3_^−^ is the most important pH buffering system in saliva. Consequently, if the ΔF508 mutation reduces secretion of bicarbonate in saliva, its pH would be expected to decrease as well. We found that the pH decreased in the whole saliva of ΔF508 mice (Figure 3A), and this acidification correlated with a dramatic, nearly 50% decrease in the HCO_3_^−^ concentration (Figure 3B) in the mutant mice.

## Discussion

Saliva is a fluid secreted primarily by the three major salivary glands, i.e. parotid, submandibular and sublingual glands. Human salivary glands typically secrete 0.5–1 liter of saliva per day in response to sympathetic and parasympathetic stimulation [29]. Fluid and electrolyte secretion involves two stages: the secretory endpiece secretes an isotonic NaCl-rich, plasma-like fluid (stage 1) which is subsequently modified as it passes through the ductal network (stage 2). Most of the NaCl is reabsorbed in the ducts, and importantly, KHCO_3_ is secreted. HCO_3_^−^ ions play a major role in buffering the pH of saliva.

Salivary glands express CFTR, an anion channel gated by an increase in intracellular cAMP. CFTR channels have been postulated to be involved in both acinar (stage 1) and ductal (stage 2) functions [15], [30]. Using the ΔF508 mouse model of cystic fibrosis, we found that the CFTR channel mediates Cl^−^ reabsorption by salivary gland ducts but fluid secretion was normal [11]. We also noted that the submandibular saliva pH in ΔF508 mice was decreased compared to their wildtype littermates. In the present study, the pH of whole saliva, which is most relevant to caries formation and progress, was also significantly reduced in the ΔF508 mouse. However, the pH of whole saliva was higher than observed previously in submandibular saliva [11]. The difference in the saliva pH between the two studies is likely the consequence of the different stimulation protocol (carbachol/isopreterenol-stimulated vs. pilocarpine-stimulated), different type of saliva collected (submandibular ductal saliva vs. whole saliva), and/or the difference in flow rate between *ex vivo* and *in vivo* approaches. Related to this last point, HCO_3_^−^ secretion is flow rate dependent.

The lower saliva pH observed in the ΔF508 mouse suggests that the ΔF508 CFTR mutation compromises the HCO_3_^−^ secretion mechanism. Consistent with this prediction, we found that the bicarbonate concentration of pilocarpine-stimulated whole saliva was severely reduced in the ΔF508 mice. HCO_3_^−^ is the most important pH buffering system in saliva, while adherence and survival of many oral bacteria are dependent on pH. Consequently, we hypothesized that *S. mutans* colonization and prevalence of carious lesions may be enhanced in the ΔF508 mouse model of cystic fibrosis. We found that the incidence of both smooth and sulcal surface caries of mandibular and maxillary molars was significantly elevated in the ΔF508 mouse. The severity of carious lesions was also dramatically elevated, increasing in most cases by an order of magnitude for both smooth and sulcal surfaces. This remarkable increase in the severity of carious lesions in the ΔF508 mouse in just five weeks exposure to a cariogenic diet is noteworthy in that it is comparable to that seen in mice that had been desalivated [28]. Nevertheless, the elevated incidence and severity of dental caries was not related to a decrease in saliva by itself because the fluid secretion volume was essentially unchanged in the ΔF508 mouse [11]. Thus, the ΔF508 CFTR mutation appears to decrease HCO_3_^−^ secretion in salivary glands, reducing the buffering capacity and pH of saliva. These phenomena would greatly affect the ability of saliva to reduce the adverse effects of acid production by *S. mutans* and other acidogenic bacteria, and thereby increase the extent of acidification within the dental plaque on the tooth surface. The persistence of this aciduric environment in the plaque's matrix leads to selection and establishment of highly acid-tolerant (and acidogenic) organisms such as *S. mutans*, and the acidic pH at plaque-tooth interface results in dissolution of enamel [2].

Another possible mechanism for the elevated incidence and severity of lesions in the CF mouse is that the ΔF508 mutation might alter the composition of the tooth enamel. However, Bronckers *et al.* found that the ameloblasts of molars were not structurally affected in mice lacking Cftr [31]. Indeed, Gawenis *et al.* failed to detect changes in the ion composition of the molars of these mice [32]. No lesions were detected in the mouse incisors probably because they erupt continuously. Consequently, considering that HCO_3_^−^ is the most important pH buffering system in saliva, and tight adherence and survival of aciduric bacteria such as *S. mutans* are enhanced at low pH values, the decrease in the HCO_3_^−^ content and pH buffering of the saliva is most likely to be at least partially responsible for the severity of lesions observed in the CF mouse. Importantly, this is the first genetic model to demonstrate a clear relationship between saliva composition and the incidence of carious lesions. Accordingly, the ΔF508 CF mouse paves the way for future studies to evaluate the complex, multifactorial relationship between host genetic factors and the pathogenesis of dental caries. The insight gained from such studies may ultimately lead to the generation of cost effective preventive agents for dental caries, the most common and costly oral infectious disease worldwide.

## Materials and Methods

### General Methods

Breeding mice were housed in micro-isolator cages with *ad libitum* access to laboratory chow and water during 12-hour light/dark cycles. Heterozygous ΔF508 Cftr (*Cftr* ^ΔF508/ΔF508^) mice on a Black Swiss/129 SvJ hybrid background were bred to generate homozygous wildtype and ΔF508 Cftr animals of either sex. This study was carried out in strict accordance with the recommendations in the Guide for the Care and Use of Laboratory Animals of the National Institutes of Health. Experimental protocol #98-232 was approved by the University of Rochester Animal Resources Committee. All surgery was performed under chloral hydrate anesthesia, and all efforts were made to minimize suffering. Reagents were obtained from Sigma (St. Louis, MO) unless otherwise specified.

### Caries Studies

Male and female breeder pairs were separated prior to birth of the pups. Pups were inoculated by swabbing the oral cavity on 3 consecutive days starting 19 days after birth with *S. mutans* cultures (strain UA159; ATCC 700610), a well-characterized cariogenic bacterium [28]. After weaning at 22 days of age, both homozygous wildtype and ΔF508 pups were fed Diet 2000 and GoLYTELY (Braintree Laboratories, MA), an oral osmotic laxative used to increase the survival of ΔF508 mice [33]. An oral swab was obtained from pups one and four days after the final inoculation. The swab was plated on selective media to verify implantation by *S. mutans* (MSB plates, Mitis salivarius + bacitracin). All wildtype and ΔF508 animals were successfully infected by *S. mutans*. After five weeks on Diet 2000, mice were killed by CO_2_ asphyxiation. Caries scores were performed as previously described on both mandibular and maxillary first and second molar teeth [28].

In a pilot study, we tested three variables: 1) are CF mice and their control littermates susceptible to infection by *S. mutans*; 2) because CF mice display intestinal absorption defects, we also tested if the cariogenic diet (diet 2000) affects CF mouse viability; and 3) is gross caries seen after a short exposure to the cariogenic diet. Mice were infected with *S. mutans* for three consecutive days, as described above, and then exposed over a 13 day period to the cariogenic diet. The pilot study confirmed that all four CF and four wildtype mice were infected by *S. mutans* and that diet 2000 did not affect the viability of the CF mice. At the end of the 13 day exposure to the cariogenic diet, photos were taken of control and CF jaws. Note that smooth surface caries is not visible in the mandibular jaws from either the control or CF mice (Figure S1). This latter result is consistent with the current understanding that dental caries is a diet bacterial disease; i.e. caries doesn't occur in the absence of appropriate infection and dietary challenge over time. Our pilot study indicates that this is also true in the CF mouse model.

To estimate the number of viable *S. mutans* cells, the mandibular jaw was aseptically dissected, transferred to 5.0 ml of sterile saline solution and sonicated as described previously [34]. The suspensions were serially diluted and plated on mitis salivarius agar plus bacitracin (for *S. mutans* counts) using an automated spiral plater (Eddy Jet, IUL Instruments, Neutec Group Inc.). Following incubation, the viable recovered cells were determined by counting colony forming units (cfu) by means of a grid plate system. In parallel, the microbial suspensions were examined using quantitative real-time PCR and propidium monoazide (PMA, Biotium Inc., Hayward, CA) to quantify only cells with intact membrane (viable cells) as detailed by Nocker *et al.* [35]. After PMA treatment, the genomic DNA of treated microbial suspensions was extracted and purified using the MasterPure DNA purification kit (Epicenter Technologies). Ten nanograms of genomic DNA per sample and negative controls (without DNA) were amplified by a MyiQ real-time PCR detection system with iQ SYBR Green supermix (Bio-Rad Laboratories, Inc., CA, USA) using *S. mutans* 16S rRNA specific primers. The primers were designed using Beacon Designer 2.0 software (Premier Biosoft International, Palo Alto, CA, USA). For *S. mutans* quantification, a standard curve based on *S. mutans* genome size (2.03 Mb) was used as described previously [36]. This standard curve was used to transform the critical threshold cycle (*Ct*) values to the relative number of *S. mutans* cells.

### *In Vivo* Whole Saliva Collection

Wildtype and ΔF508 mutant animals were anesthetized with chloral hydrate (400 mg/kg body weight, i.p.). Prior to saliva collection a tracheal tube was placed to maintain a patent airway during stimulation. Secretion was initiated by the injection of the muscarinic agonist pilocarpine HCl (10 mg/kg, i.p.). Whole saliva was collected by gravity into 1.5 mL plastic eppendorf tubes.

### Saliva Ion Composition

The concentration of bicarbonate was determined as described by the manufacturer (Diazyme Laboratories, Poway, CA). Saliva pH was measured immediately after collection with a pH-sensitive electrode (Thermo Scientific, Beverly, MA).

### Statistical Analysis

Graphs showed in figures 1, 2 and 3 are presented as the mean ± S.E. and the statistical significance was determined using Student's *t* test with Origin 7.0 Software (OriginLab, Northampton, MA). *p* values of less than 0.05 were considered statistically significant.

## Supporting Information

Figure S1 **Pilot study for dental caries in wildtype and ΔF508 mice.** Lingual (panels A and C) and buccal (panels B and D) views of representative mandibular jaws from wildtype (WT, panels A and B) and mutant mice (ΔF508, panels C and D) show that no visible lesions were observed after 13 days exposure to a cariogenic diet.
(TIF)
