# Supplementary figures and images for: Elevated Incidence of Dental Caries in a Mouse Model of Cystic Fibrosis
Source: PLoS One. 2011 Jan 31;6(1):e16549. doi: 10.1371/journal.pone.0016549 (PMC3031584; doi:10.1371/journal.pone.0016549)

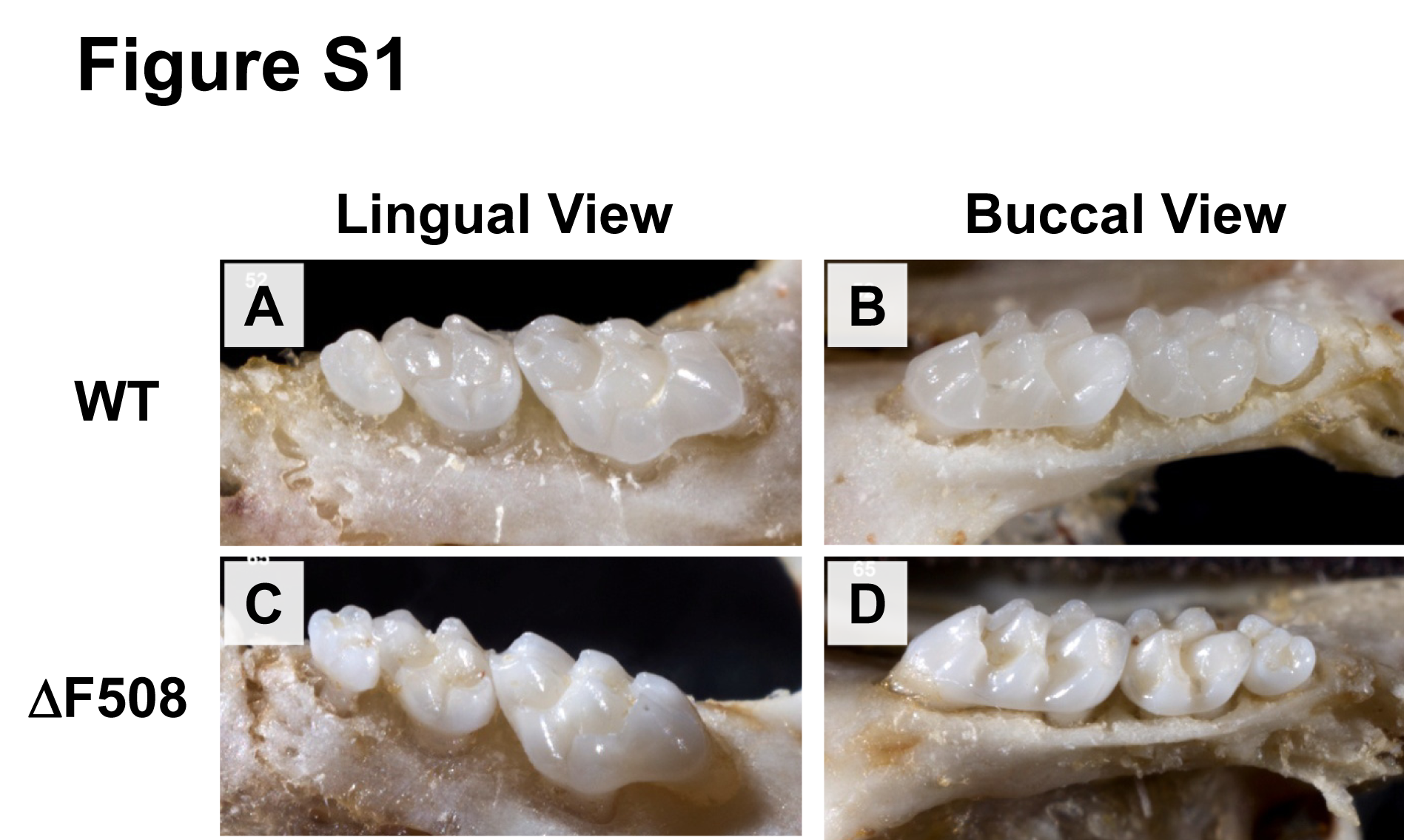

Supplement: Figure S1 — Pilot study for dental caries in wildtype and ΔF508 mice. Lingual (panels A and C) and buccal (panels B and D) views of representative mandibular jaws from wildtype (WT, panels A and B) and mutant mice (ΔF508, panels C and D) show that no visible lesions were observed after 13 days exposure to a cariogenic diet. (TIF) [file pone.0016549.s001.tif]
